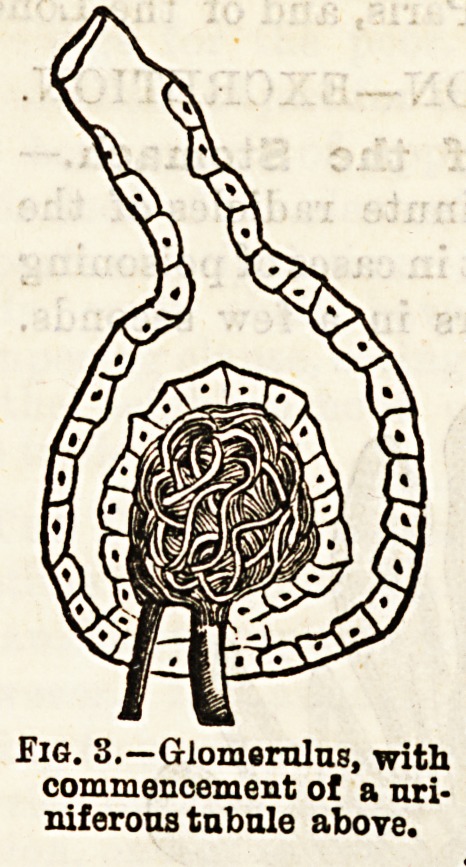# Diet in Disease

**Published:** 1892-12-17

**Authors:** Ernest Hart

**Affiliations:** Bachelier-ès-Sciences-es-Lettres (rèstreint), formerly Student of the Faculty of Medicine of Paris, and of the London School of Medicine for Women


### Diet in Disease.

By Mrs. Ernest Hart, Bachelier-ès-Sciences-es-Lettres (rèstreint), formerly Student of the Faculty of Medicine of Paris, and of the London School of Medicine for Women.

### X.—DIGESTION—ABSORPTION—EXCRETION.

**Absorption by the Veins of the Stomach.**—The absorption of fluids by the minute radicles of the veins of the stomach is so rapid that in cases of poisoning by hydrocyanic acid, death occurs in a few seconds.

Fig. 1.—Villi on a fold of the jejunum.

There is no doubt that the gastric juice which is poured out in such large quantities during the process of digestion in the stomach is absorbed together with the albumose, the dissolved salts, and the sugar derived from the food. In this way the system is relieved from the excessive drain which would be thrown upon it, if the whole of the gastric juice required for the digestion of a meal had to be manufactured and thrown out anew each time. The absorption of the gastric juice secreted in the process of absorption, in conjunction with the soluble albumoses or peptones, is one of the most important things to remember in the dietetic treatment of dyspepsia; for, if we could succeed, by the rapid absorption of fluids by the veins of the stomach, in providing the peptic glands with the material out of which to manufacture pepsin, the digestive process would be immediately aided in cases of atonic dyspepsia.

**Absorption from the Intestines.—The Villi and Lacteals.**—If a portion of the small intestine of any vertebrate animal be opened, washed, and floated in water, the internal surface will be seen to resemble that of velvet, and to be covered with a countless number of minute projections, or *villi* (Fig. 1). I have already stated that the mucous membrane of the small intestine consists of a vast number of tubular depressions or glands. It is between these glands and on their edges or surfaces that the villi of the intestine project. It will thus be seen that the area of the internal wall of the intestine is enormously increased by the alternate dippings and elevations of its surface. The part which folds in or dips, forming the tubular glands, is concerned in secretion, and the part which is elevated, forming the villi, is concerned in absorption. The construction of a villus is as follows: (Fig. 2.) In the centre is found an inverted tube or canal, closed at one end, the walls of which are formed of their transparent epithelial cells; this is the blind end of a lacteal. Closely covering it is a fine network of extremely small blood vessels. The external surface of the villus is covered by a single layer of columnar epithelial cells closely set together. The absorption of fat by the lacteals takes place in the following manner: The minute globules of fat which have been emulsified by the action of the pancreatic juice, the bile, and the intestinal juice, pass through and between the epithelial cells which form the outer lining of the villus, and also through the transparent thin wall of the lacteals. The lacteals communicate with a fine network of lymphatic vessels which ramify in the surface of the mesentery, or membrane to which the intestines are attached. Along these vessels, which are abundantly provided with valves to prevent a backward current, the oil-globules, absorbed from the digested food, slowly pass, till gathered into a larger vessel called the thoracic duct. This duct passes upwards beside the vertebral column, and pours its contents into the left jugular vein in the neck.

**Absorption by the Capillary Blood Vessels of the Intestine** is, however, much more important than by the lacteals. It is seen from the structure of the villi that there is only a single layer of epithelial cells intervening between the digested fluid foods in the intestinal canal and the extensive surface of the capillary vessels. Absorption, therefore, of all solvent and fluid matters from the intestine into the veins of the villi, takes place easily and rapidly. The intestinal juice which is poured out in such abundance during digestion is also re-absorbed by the blood vessels of the villi.

Fig. 2.—Minute structure of villi, *l*, lacteal; *v*, radicles of veins.

**The Portal Circulation.**—The blood now laden with the products of digestion passes from the venous capillaries of the stomach and intestines to the blood vessels of the mesentery. These pour their contents into a large vessel called the portal vein, which conducts the blood direct to the liver. It will be seen that by this arrangement the albumose derived from albuminous foods, the glucose derived from the starch and sugar, together with the various salts held in solution, are carried direct to the liver by the portal vein, there to be elaborated into the substances necessary for nutrition. The glucose is converted by the action of the liver cells into glycogen, and is, it is asserted, ultimately restored to the circulation in the form of glucose, to be probably burnt up in the tissues in the processes of metabolism or tissue change, and the albumose is, after passing through the liver, returned to the circulation in the form of blood albumen.

Most of the fatty particles of the food are absorbed by the lacteals, and enter the general current of the circulation by the thoracic duct pouring its contents into the left jugular vein. The jugular vein leads into the superior vena cava, which conducts the blood into the right side of the heart, from which it is pumped by the pulsation of the heart into the lungs. In the lungs the fatty particles with which the blood is charged after a meal entirely disappear, and are probably burnt up in the process of the maintenance of the body heat.

Thus in passing through the two great separative and constructive organs of the body; the lungs and the liver, the venous blood, charged both with the products of decomposition (carbonic acid gas), and with the materials for repair, undergoes such changes by casting out the products of tissue destruction, and by modifying the materials of reconstruction, that it issues both from the lungs and the liver in a renovated condition, and charged with those materials which are necessary for the growth and repair of the tissues. The blood as it issues from the lungs is carried by the pulmonary artery, and from the liver by the hepatic artery, into the aorta, and is conveyed by the circulation into the furthermost parts of the body, where it nourishes the tissues. But the lungs and the liver have not done all that is necessary for the scavenging and renovation of the blood. After leaving the liver it passes all through the tissues, is again collected together by the veins, and passed on to the right side of the heart. It is thence pumped into the lungs, where it parts with its carbonic acid gas, and receives a new and revivifying supply of oxygen. Passing again into the heart, it is pumped from the left side into the aorta, thence to be distributed to the body. But though apparently cleansed by its passage through the lungs, it is still laden with the products of the decomposition, incomplete oxidation, or retrogressive changes which have taken place in the tissues in the course of tissue change or growth, and in the production of energy. These effete products are more particularly those resulting from the incomplete oxidation of albumen, which are found in the blood in the form of urea and uric acid. As these are most deleterious in their effects, and even poisonous in their action if allowed to circulate in the blood, it becomes a matter of the greatest importance to get rid of them. This is accomplished by the action of the kidneys.

**The Kidneys** are two bean-shaped bodies, which lie at the back of the abdominal cavity, on either side of the vertebral column in the lumbar region. The arteries which conduct the blood to them come off at right angles to the aorta, from which arrangement it is obvious that the blood passes with considerable force into the kidneys from the main channel of the circulation. Within the kidney the artery at once divides into a number of various vessels which end in what is called a glomerulus. In the glomerulus the vessel breaks up into a great number of finer vessels folded one upon another in a tangled ball. The arteriole communicates with a vein similarly constructed and arranged, but of smaller calibre, hence the return of the blood from the glomerulus is somewhat hindered. This convoluted ball of blood vessels is pushed into the globe-like distension of a fine tube. (Fig. 3.) The delicate transparent double walls of the sack-like end of the tube envelope the glomerulus on every side.

Fig. 3.—Glomerulus, with commencement of a uriniferous tubule above.

Here we obviously have all that is necessary for the process of filtration: blood carried at high tension from the full current of the circulation suddenly brought almost to a condition of stasis in the tangle of the glomerulus, and a bag or filter furnished with a conducting tube in immediate contact with the distended blood vessels. What happens is that the watery constituents of the blood, together with the urea and other extractive and colouring matters, are filtered from the capillary vessels into the sack-like termination of the uriniferous tubule. In a state of health the albumen and fibrin of the blood do not pass this filter. The urine thus excreted from the blood in the glomerulus passes by a series of looped vessels into a single tube which opens into a basin-like cavity called the pelvis of the kidney. The fluid which is being constantly forced out from the uriniferous tubuli is finally conveyed from both kidneys by long, narrow muscular tubes called the ureters to the bladder, which is emptied at will.

**To Recapitulate.**—I.—1. Starchy foods are converted into glucose in the mouth by the action of the saliva, and in the duodenum by the action of the pancreatic juice.

2. Albuminous foods are converted into soluble albumoses or peptones by the action of the pepsin of the gastric juice acting in an acid medium, and by the trypsin of the pancreatic juice acting in an alkaline or neutral medium.

3. Fats are emulsified in the intestines by the action of the pancreatic juice, the bile, and the intestinal juice.

II.—1. Albumose is absorbed by the venous radicles of the stomach and intestines, and carried by the portal vein to the liver.

2. Glucose is absorbed by the capillaries of the villi, and carried by the mesenteric veins to the portal vein, and thence direct to the liver.

3. Emulsified fats are absorbed by the lacteals, and are carried by the thoracic duct to the left jugular vein.

III.—1. Albumose is converted by the liver into albumen and is present in the blood in the form of blood serum and fibrin.

2. Glucose is converted by the action of the liver into glycogen, and is stored there for use in the economy.

3. Fats are burnt off in the lungs and in the tissues in the production of body heat. Fat is also stored up in the tissues for future use.

The various digestive juices are re-absorbed during and after the process of digestion.

The execretory products of digestion are the bile excreted by the liver; the urine, containing urea excreted by the kidneys, and the fæces, containing the indigestible and undigested remnants of food, broken down cells, masses of bacilli which flourish in the intestine, and the colouring matters of the bile, which are excreted by the rectum.

Any abnormal divergence from the long and complicated process of digestion will give rise to many conditions of ill-health and disease, to dyspepsia, gout, diabetes, &c. All these will be considered in turn, as well as their alleviation and possible cure by means of diet. In the following chapters I propose to illustrate the principles of diet and the facts of digestion already described, by considering typical foods both for health and disease.

## Figures and Tables

**Fig. 1. f1:**
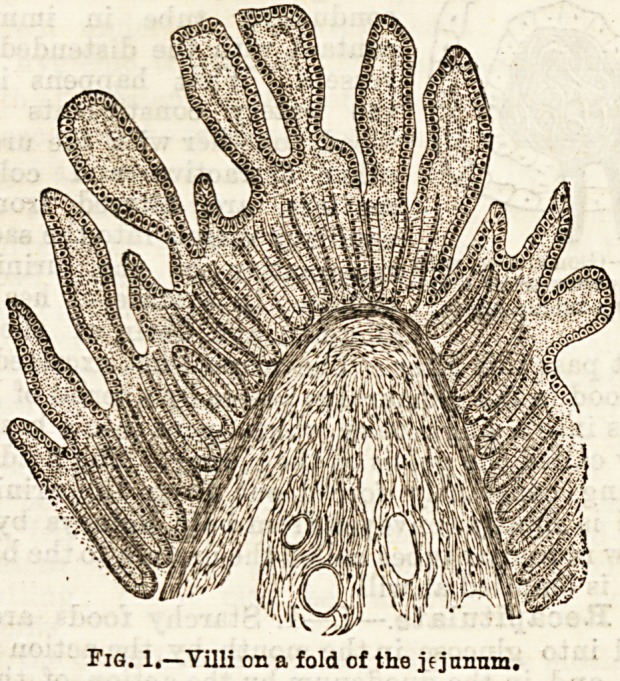


**Fig. 2. f2:**
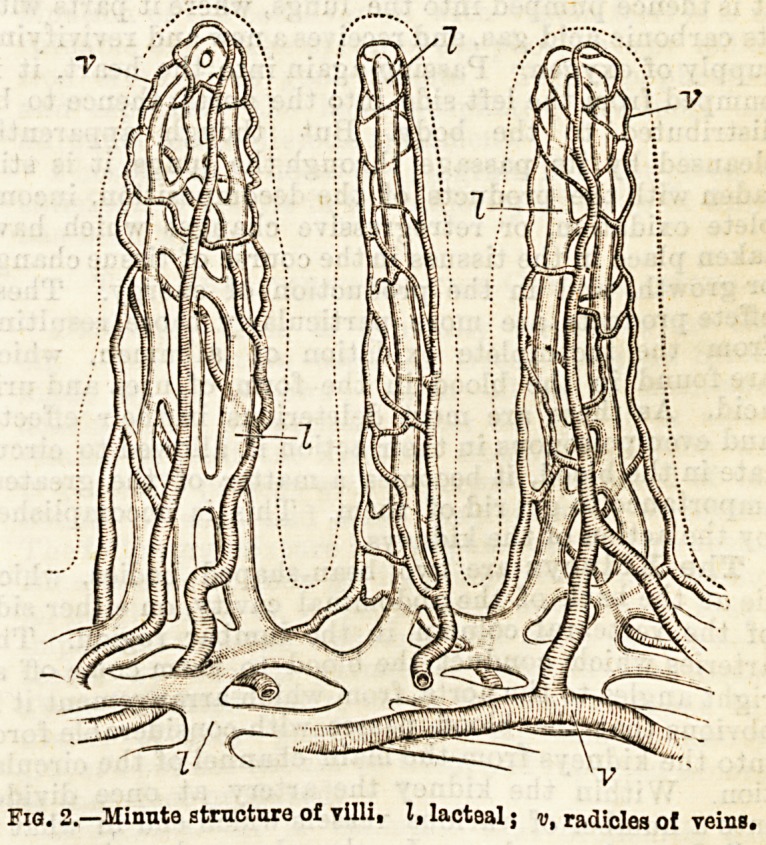


**Fig. 3. f3:**